# D-galactose induces senescence of glioblastoma cells through YAP-CDK6 pathway

**DOI:** 10.18632/aging.103819

**Published:** 2020-09-29

**Authors:** Xingxing Xu, Xiya Shen, Wenjin Feng, Danlu Yang, Lingting Jin, Jiaojiao Wang, Mianxian Wang, Zhang Ting, Feng Xue, Jingjing Zhang, Chaobo Meng, Roumeng Chen, Xinru Zheng, Leilei Du, Lina Xuan, Ying Wang, Tian Xie, Zhihui Huang

**Affiliations:** 1 School of Basic Medical Sciences, Wenzhou Medical University, Wenzhou 325035, Zhejiang, China; 2 Key Laboratory of Elemene Anti-Cancer Medicine of Zhejiang Province and Holistic Integrative Pharmacy Institutes, and Department of Neurosurgery of Affiliated Hospital, Hangzhou Normal University, Hangzhou 311121, China; 3 Department of Orthopedics (Spine Surgery), The First Affiliated Hospital of Wenzhou Medical University, Wenzhou 325035, Zhejiang, China; 4 Zhejiang Sinogen Medical Equipment Co., Ltd, Wenzhou, 325000, Zhejiang, China; 5 Department of Transfusion Medicine, Zhejiang Provincial People’s Hospital of Hangzhou Medical College, Hangzhou 310053, China; 6 Department of Neurobiology, Key Laboratory of Medical Neurobiology, Ministry of Health of China, School of Medicine, Zhejiang University, Hangzhou,310058, China

**Keywords:** D-galatose, cellular senescence, glioblastoma, YAP, CDK6

## Abstract

Treatment of glioblastoma using radiotherapy and chemotherapy has various outcomes, key among them being cellular senescence. However, the molecular mechanisms of this process remain unclear. In the present study, we tested the ability of D-galactose (D-gal), a reducing sugar, to induce senescence in glioblastoma cells. Following pretreatment with D-gal, glioblastoma cell lines (C6 and U87MG) showed typical characteristics of senescence. These included the reduced cell proliferation, hypertrophic morphology, increased senescence-associated β-galactosidase activity, downregulation of Lamin B1, and upregulation of several senescence-associated genes such as p16, p53, and NF-κB. Furthermore, our results showed that D-gal was more suitable than etoposide (a DNA-damage drug) in inducing senescence of glioblastoma cells. Mechanistically, D-gal inactivated the YAP-CDK6 signaling pathway, while overexpression of YAP or CDK6 could restore D-gal-induced senescence of C6 cells. Finally, metformin, an anti-aging agent, activated the YAP-CDK6 pathway and suppressed D-gal-induced senescence of C6 cells. Taken together, these findings established a new model for analyzing senescence in glioblastoma cells, which occurred through the YAP-CDK6 pathway. This is expected to provide a basis for development of novel therapies for the treatment of glioblastoma.

## INTRODUCTION

Glioblastoma (GBM; WHO grade IV) is an aggressive form of brain tumor that accounts for about 40-50% of all primary intracranial tumors [1]. Standard therapeutic strategies for management of GBM include surgery, radiotherapy, and chemotherapy. In the past, to develop better treatments of GBM, most studies focused on the proliferation and migration of GBM cells [2, 3], however, effective treatments are still not available. Despite the advancement in treatments such as radiotherapy and chemotherapy in combination with surgical resection, prognosis of GBM patients remains unsatisfactory. In fact, a median survival time of about 15 months has been reported in GBM cases, while the 5-year survival rate remains at less than 5% [4]. Therefore, finding an effective strategy to improve the therapeutic effects on malignant GBM has become one of the most imperative and urgent necessities in neurosurgery.

Radiotherapy and chemotherapy have been found to cause senescence of GBM cells [5, 6]. Senescent cells exhibit some common features that include large, flat, and vacuolized cell morphology, stagnation of cell growth, increased senescence-associated β-galactosidase activity, accumulation of p16 and p53, decreased expression of Lamin B1, and appearance of senescence-associated secretory phenotype (SASP) [7–12]. Some studies have shown that senescence of tumor cells confers a protective mechanism for survival by entering the dormant period, evading treatment, and contributing to tumor recurrence [13, 14]. In fact, persistent senescence is considered as detrimental. However, transient senescence can initiate a beneficial process which triggers elimination of senescent cells, probably through the inflammatory microenvironment [15, 16]. Therefore, reversing persistent senescence of GBM cells or inducing their transient senescence may be an effective strategy for suppression and treatment of GBM [7, 16]. Nevertheless, the molecular mechanisms that regulate this process are poorly understood.

D-gal, which is used to induce ageing in animals [17, 18], can also be used to prepare senescent models of some cell types, including astrocytes [19] and lens epithelial cells [20]. Functionally, D-gal induces cell senescence through multiple mechanisms such as glutamine synthetase signaling [19], or by disturbing autophagy flux and mitochondrial functions [20]. However, to date, it is not known whether D-gal can induce senescence of GBM cells. Moreover, since the underlying mechanisms of cellular senescence may vary under different conditions, it is imperative to explore the mechanisms in D-gal-induced GBM cell senescence.

Yes-associated protein (YAP) is a key effector in the Hippo signaling, a conserved classical pathway formed by the kinase cascade that regulates stem cell self-renewal, tissue regeneration and organ size. When this pathway is inactive, Yap/Taz is translocated into the nucleus, thereby initiating expression of pro-proliferation and pro-survival genes, and promoting cell proliferation [21]. In addition, Cyclin-dependent kinase 6 (CDK6) is a kind of cyclin-dependent serine-threonine kinase that is required for G1-S phase cell cycle progression and cell proliferation [22]. CDK4/6 signaling has been linked to suppression of senescence via expression of the FOXM1 transcription factor [23]. Moreover, CDK6 has been identified as a novel downstream target of the YAP-TEAD transcriptional active complex, where it mediates senescence of IMR90 cells [24]. However, the role played by YAP-CDK6 pathway in senescence of GBM cells remains unknown.

In the present study, we examined the role and mechanisms of D-gal in senescence of GBM cells and found that this compound could induce senescence. The method reported herein is simple and fast, with high success rate and shows good effects. Moreover, we found that D-gal induced senescence of GBM cells through the YAP-CDK6 pathway. Taken together, the findings in this model can provide a novel basis for the treatment of GBM.

## RESULTS

### D-gal decreases cell viability but does not induce apoptosis of GBM cells

**Figure 1:**
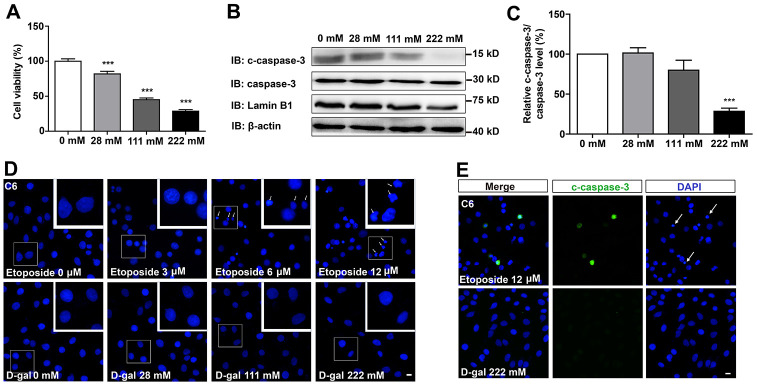
**D-gal decreased cell viability, but did not induce apoptosis of GBM cells.** (**A**) The effects of D-gal (treated for 8 d) on C6 cell viability as detected by CCK8 (n=3). (**B**) Western blot detected the expression of c-caspase-3, caspase-3 and Lamin B1 in cells treated with 0 mM, 28 mM, 111 mM and 222 mM D-gal for 8 d. (**C**) Quantification of c-caspase-3/caspase-3 protein level as shown in (B) (n=4). “Relative c-caspase-3/total caspase-3 level” of the 0 mM group = [c-caspase-3/total caspase-3 (0 mM group)]/[c-caspase-3/total caspase-3 (0 mM group)] ×100% = 100%; “Relative c-caspase-3/total caspase-3 level” of other D-gal-treated groups = [c-caspase-3/total caspase-3 (other D-gal-treated groups)]/[c-caspase-3/total caspase-3 (0 mM group)] ×100%. (**D**) DAPI staining of C6 cells treated with 0 μM, 3 μM, 6 μM, 12 μM etoposide for 1 d, and recovered for 4 d or with D-gal at 0 mM, 28 mM, 111 mM and 222 mM for 8 d. The white arrows indicated apparently dead cells. (**E**) Immunostaining of c-caspase-3 in cells treated with 12 μM etoposide for 1 d, and recovered for 4 d, or with D-gal at 222 mM for 8 d. The white arrows indicated apparently dead cells. Scale bars, 20 μm. Data shown are mean ± s.e.m. ^***^P < 0.001.

To examine the effects of D-gal on GBM cells, C6 cells were treated with different concentrations of the compound for 8 days. As shown in Figure 1A, D-gal reduced cell viability in a concentration-dependent manner. Particularly, 222 mM of D-gal resulted in a more significant reduction of C6 cell viability than other concentrations. Surprisingly, results from western blot analysis showed that cleaved-caspase-3 (c-caspase-3)/caspase-3 level was significantly decreased in D-gal-treated groups, compared to the control group (Figure 1B, 1C), indicating that the reduction in cell viability by D-gal was not due to apoptosis of C6 cells, but owing to anti-apoptosis effects observed in these cells. In addition, immunostaining of the nucleus and c-caspase-3 also confirmed that D-gal treatment did not cause apparent apoptosis of C6 cells, whereas etoposide (a DNA-damage drug) treatment could induce significant apoptosis of these cells (Figure 1D, 1E). Taken together, these results suggest that D-gal decreases cell viability, not due to induction of apoptosis in GBM cells.

### D-gal induces premature senescence of GBM cells

**Figure 2:**
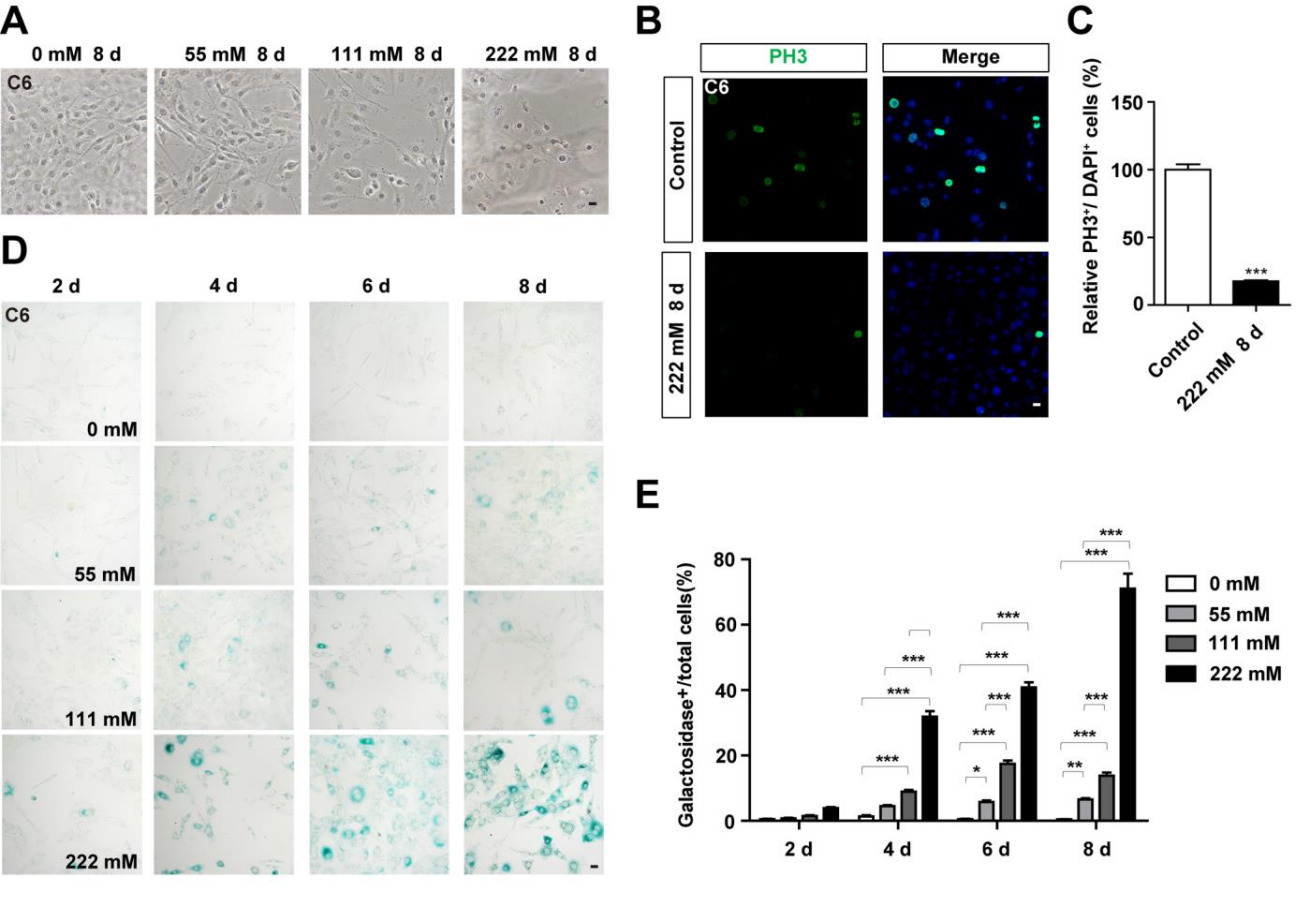
**D-gal treatment induced premature senescence of GBM cells.** (**A**) Representative images of C6 cells (in the bright field) treated with D-gal at 0 mM, 28 mM, 111 mM and 222 mM for 8 d. (**B**) Immunostaining analysis of PH3 (green) in control cells and in C6 cells treated with 222 mM D-gal for 8 d. (**C**) Quantitative analysis of the percentage of PH3^+^ cells over total cells as shown in (**B**) (n=15). (**D**) Representative images showing β-galactosidase staining in C6 cells treated with D-gal at 0 mM, 28 mM, 111 mM and 222 mM concentrations for 2, 4, 6 and 8 d. (**E**) Quantification of the percentage of β-galactosidase^+^ C6 cells over total cells as shown in (**D**) (n=15). Scale bars, 20 μm. Data shown are mean ± s.e.m. ^*^P < 0.05, ^**^P < 0.01, ^***^P < 0.001.

To examine whether D-gal can induce the senescence of GBM cells, C6 cells were treated with different concentrations of the compound for 8 days. As expected, the growth potential of treated cells was significantly inhibited in a concentration dependent manner, relative to the control (Figure 2A). Moreover, C6 cells exposed to 111 mM or 222 mM D-gal displayed a hypertrophic, thick and flattened morphology, which was the characteristic of cellular senescence (Figure 2A). Since cell cycle arrest and inhibition of proliferation are hallmarks of cellular senescence [7], we performed immunostaining of PH3 and Ki67 (both are markers for cell proliferation), and found a reduction in PH3 and Ki67 positive cells, indicating that D-gal treatment inhibited the proliferation of C6 cells (Figure 2B, 2C, Supplementary Figure 1A, 1B). According to previous reports, the most commonly used approach for detecting senescent cells is β-galactosidase staining [9]. Indeed, as shown in Figure 2D, 2E, D-gal treatment significantly increased β-galactosidase positive cells in a concentration- and time-dependent manner. Particularly, D-gal at a concentration of 222 mM for 8 days was optimal for induction of senescence in C6 cells, and was used for subsequent experiments. Taken together, these results suggest that D-gal induces premature senescence of GBM cells.

### D-gal induces changes of senescence makers in GBM cells

**Figure 3:**
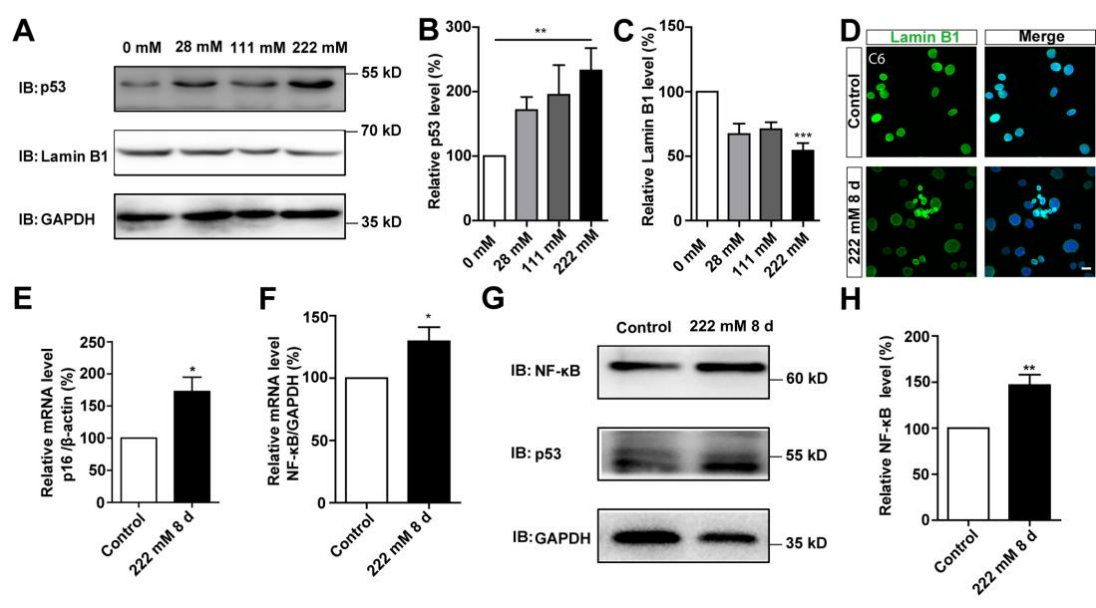
**D-gal induced changes of senescence makers in C6 cells.** (**A**) Western blot detected the expression of p53 and Lamin B1 in C6 cells treated with 0 mM, 28 mM, 111 mM, and 222 mM D-gal for 8 d. (**B**, **C**) Quantification of p53 (n=7) and Lamin B1 (n=5) expression as shown in (**A**). (**D**) Immunostaining analysis of Lamin B1 (green) in control and senescent C6 cells (treated with 222 mM D-gal for 8 d). (**E**) qPCR analysis of p16 mRNA level in control cells and senescent C6 cells (treated with 222 mM D-gal for 8 d) (n=3). (**F**) qPCR analysis of NF-κB mRNA level in control cells and senescent C6 cells (treated with 222 mM D-gal for 8 d) (n=3). (**G**) Western blot detected the expression of NF-κB and p53 in control cells and senescent C6 cells (treated with 222 mM D-gal for 8 d). (**H**) Quantification of NF-κB expression as shown in (**G**) (n=12). Scale bars, 20 μm. Data shown are mean ± s.e.m. ^*^P < 0.05, ^**^P < 0.01, ^***^P < 0.001.

Next, we performed biochemical and cellular analyses to further understand D-gal-induced senescence in GBM cells. During the ageing process, cells show increased expression of p53 and p16 [10], and decreased expression of Lamin B1 [11]. Expression of p53 and Lamin B1 were therefore detected in C6 cells treated with different concentrations of D-gal for 8 days. As expected, analysis of western blots showed a significant increase in p53 protein levels in D-gal-treated C6 cells, whereas those of Lamin B1 significantly decreased, in a concentration-dependent manner (Figure 3A–3C). In addition, as shown in Figure 3D, immunostaining showed that the level of Lamin B1 was decreased in D-gal-treated C6 cells. Conversely, the mRNA level of p16 was significantly increased in D-gal-treated C6 cells (Figure 3E). Studies have shown that presence of SASP is a common result of cell senescence [7, 12], and is mediated by multiple inflammatory factors, such as the transcription factor NF-κB [25]. In this study, we evaluated expression of NF-κB and found significantly elevated levels of both its mRNA and protein after D-gal treatment (Figure 3F–3H). To investigate whether the appearance of the senescence of GBM could be reversed or eliminated, D-gal was removed after cellular senescence, we found that C6 cells could recover from senescence gradually (Supplementary Figure 2). This indicates that the type of senescence induced by D-gal in GBM cells is transient, and can be reversed. It is therefore belongs to beneficial senescence, and may be an effective strategy for GBM suppression and treatment. Taken together, these results further suggest that D-gal induces the senescence of GBM cells.

### D-gal induces senescence of other GBM cell line

**Figure 4:**
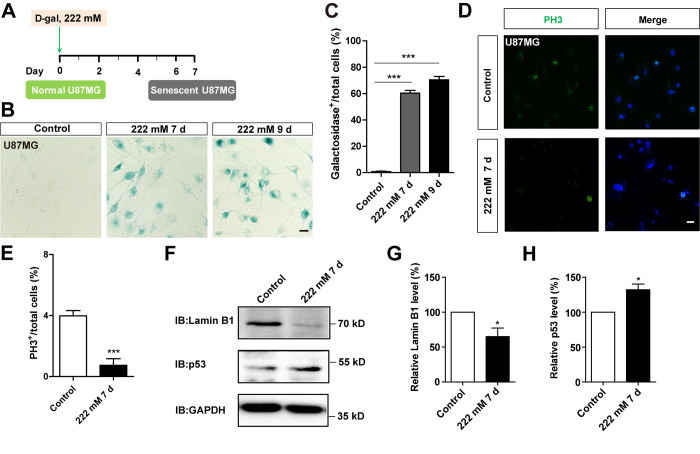
**D-gal induced premature senescence of other GBM cells.** (**A**) A schematic illustration of D-gal-induced senescence of U87MG cells. (**B**) Representative images showing β-galactosidase staining in control U87MG cells and U87MG cells treated with D-gal at 222 mM for 7 and 9 d. (**C**) Quantification of the percentage of β-galactosidase^+^ U87MG cells over total cells as shown in (**B**) (n=15). (**D**) Immunostaining of PH3 (green) in control U87MG cells and senescent U87MG cells. (**E**) Quantitative analysis of the percentage of PH3^+^ cells over total cells in as shown (**D**) (n=15). (**F**) Western blot detected the expression of Lamin B1 and p53 proteins in control U87MG cells and U87MG cells treated with 222 mM D-gal for 7 d. (**G**, **H**) Quantification of Lamin B1 (n=5) and p53 (n=5) expression as shown in (**F**). Scale bars, 20 μm. Data shown are mean ± s.e.m. ^*^P < 0.05, ^***^P < 0.001.

To determine whether D-gal-induced C6 cell senescence can be applicable to other GBM cell lines, the aforementioned experiments were performed in U87MG cells. As expected, we found that D-gal could also induce senescence of U87MG cells. Treatment of these cells with 222 mM D-gal for 7 days or 9 days resulted in a significant increase in the percentage of β-galactosidase-positive U87MG cells, at 60.36 ± 2.020 % and 70.43 ± 2.535 %, respectively (Figure 4A–4C). In addition, as shown in Figure 4D, 4E and Supplementary Figure 1C, 1D, the percentage of PH3 or Ki67 positive cells was significantly decreased, indicating that D-gal treatment reduced the proliferation of U87MG cells. Western blots further showed a significant elevation of p53 protein levels in D-gal-treated U87MG cells, whereas those of Lamin B1 showed a significant decrease (Figure 4F–4H). However, D-gal, even at high concentration, failed to induce senescence of SH-SY5Y cells, a neuroblastoma cell line (Supplementary Figure 3A, 3B), although etoposide also failed to induce senescence of SH-SY5Y cells (Supplementary Figure 3C, D). Taken together, these results suggest that D-gal is capable of inducing senescence of another GBM cell line, U87MG cells, but not SH-SY5Y cells.

### D-gal more effectively induces senescence of GBM cells than etoposide

**Figure 5:**
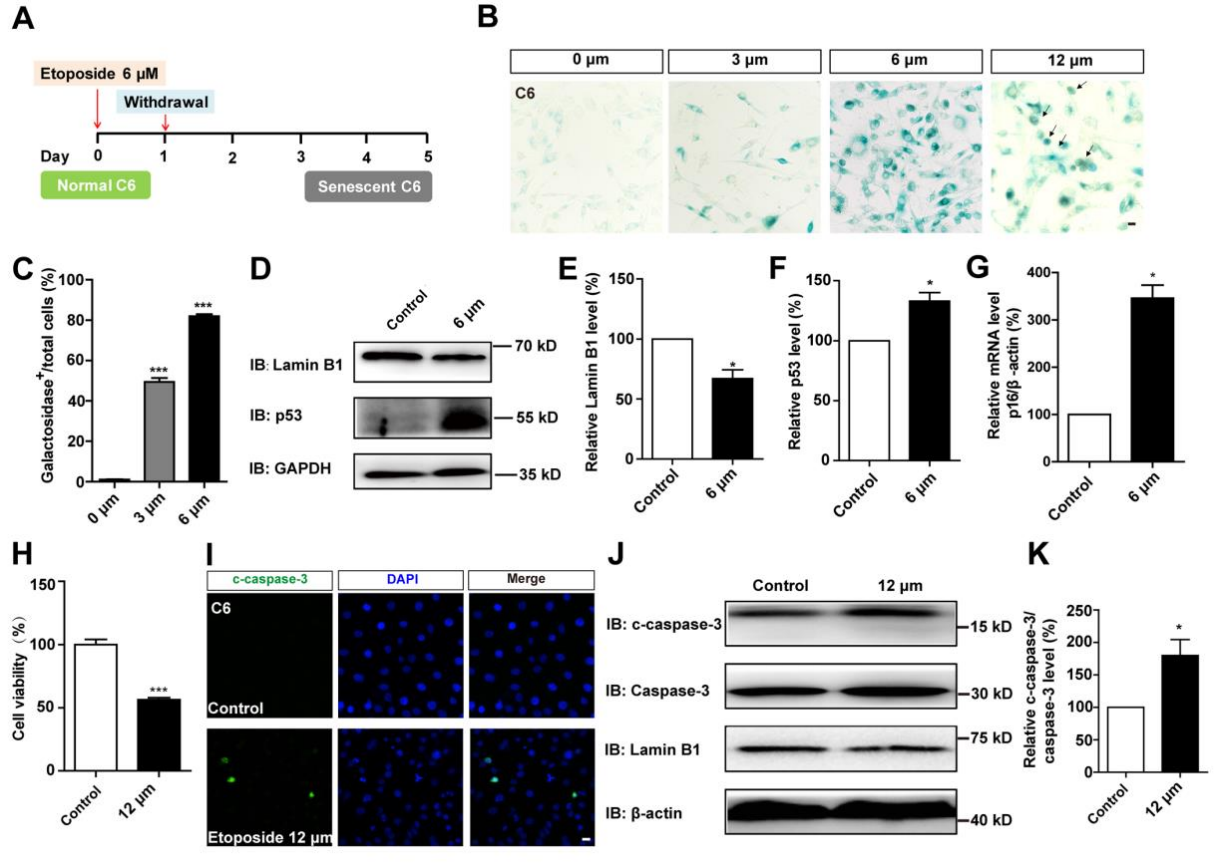
**D-gal more effectively induced senescence of GBM cells than etoposide.** (**A**) A schematic diagram showing etoposide-induced C6 cell senescence. (**B**) Representative images showing β-galactosidase staining in C6 cells treated with etoposide at 0 μM, 3 μM, 6 μM, 12 μM for 1 d, and recovered for 4 d. The black arrows indicated apparently dead cells. (**C**) Quantification of the percentage of β-galactosidase^+^ C6 cells over total cells as shown in (**B**) (n=15). (**D**) Western blot detected the expression of Lamin B1 and p53 in control cells and C6 cells treated with etoposide at 6 μM for 1 d, and recovered for 4 d. (**E**, **F**) Quantification of Lamin B1 and p53 expression as shown in (**D**) (n=5). (**G**) qPCR analysis for p16 mRNA level in control cells and senescent C6 cells (treated with etoposide at 6 μM for 1 d, and recovered for 4 d) (n=3). (**H**) The effects of etoposide (treated with 12 μM etoposide for 1 d, and recovered for 4 d) on the viability of C6 cells as detected by CCK8 assay (n=3). (**I**) Immunostaining of c-caspase-3 in control cells and senescent C6 cells (treated with 12 μM etoposide for 1 d, and recovered for 4 d). (**J**) Western blot detected the expression of c-caspase-3, caspase-3, and Lamin B1 in control cells and senescent C6 cells (treated with etoposide at 12 μM for 1 d, and recovered for 4 d). (**K**) Quantification of c-caspase-3/caspase-3 level as shown in (**J**) (n=4). Scale bars, 20 μm. Data shown are mean ± s.e.m. ^*^P < 0.05, ^***^P < 0.001.

To compare the effects of D-gal-induced senescence in GBM cells with other drugs, C6 cells were treated with etoposide, a DNA-damage drug that is also used to induce senescence in other systems, such as mouse embryonic fibroblast cells (MEFs), mouse melanoma cells (B16F10), and human foreskin fibroblast cells (BJ) [26]. C6 cells were exposed to different concentrations of etoposide for 1 day, then recovered for another 4 days. Our results showed that 6 μM etoposide was the optimum concentration for inducing senescence (Figure 5A–5C). Western blot analysis further showed that the protein level of Lamin B1 was reduced, while p53 was elevated in etoposide-treated C6 cells (Figure 5D–5F). Moreover, qPCR analysis showed that the mRNA level of p16 was significantly increased in these etoposide-treated C6 cells (Figure 5G). However, the senescent cells were accompanied by apparent cell apoptosis, even at 6 μM (Figure 1D, 1E). Cells exposed to higher concentrations of etoposide exhibited obvious signs of toxicity, including decreased viability (Figure 5H) and increased apoptosis (Figure 5I–5K and Figure 1E). Therefore, D-gal was more effectively to induce the senescence of GBM cells than etoposide.

### D-gal induces senescence of GBM cells by inactivating the YAP-CDK6 pathway

**Figure 6:**
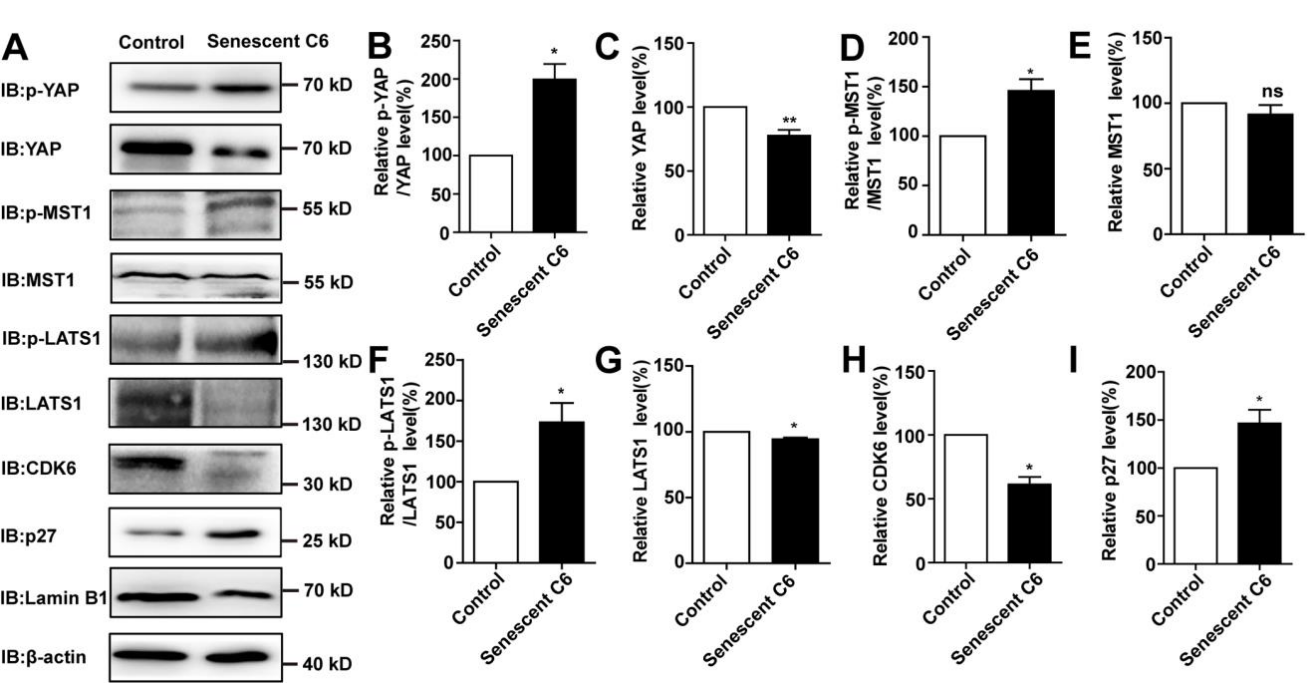
**D-gal induced senescence of GBM cells by inactivating YAP-CDK6 pathway in a Hippo pathway dependent manner.** (**A**) Western blot detected the protein expression of p-YAP, YAP, p-MST1, MST1, p-LATS1, LATS1, CDK6, p27^kip1^, and Lamin B1 in control and senescent C6 cells (treated with 222 mM D-gal for 8 d). (**B**–**I**) Quantification of protein levels of p-YAP/YAP, YAP, p-MST1/MST1, MST1, p-LATS1/LATS1, LATS1, CDK6 and p27^kip1^ (n=3) level as shown in (**A**). Data shown are mean ± s.e.m. ^*^P < 0.05, ^**^P < 0.01.

**Figure 7:**
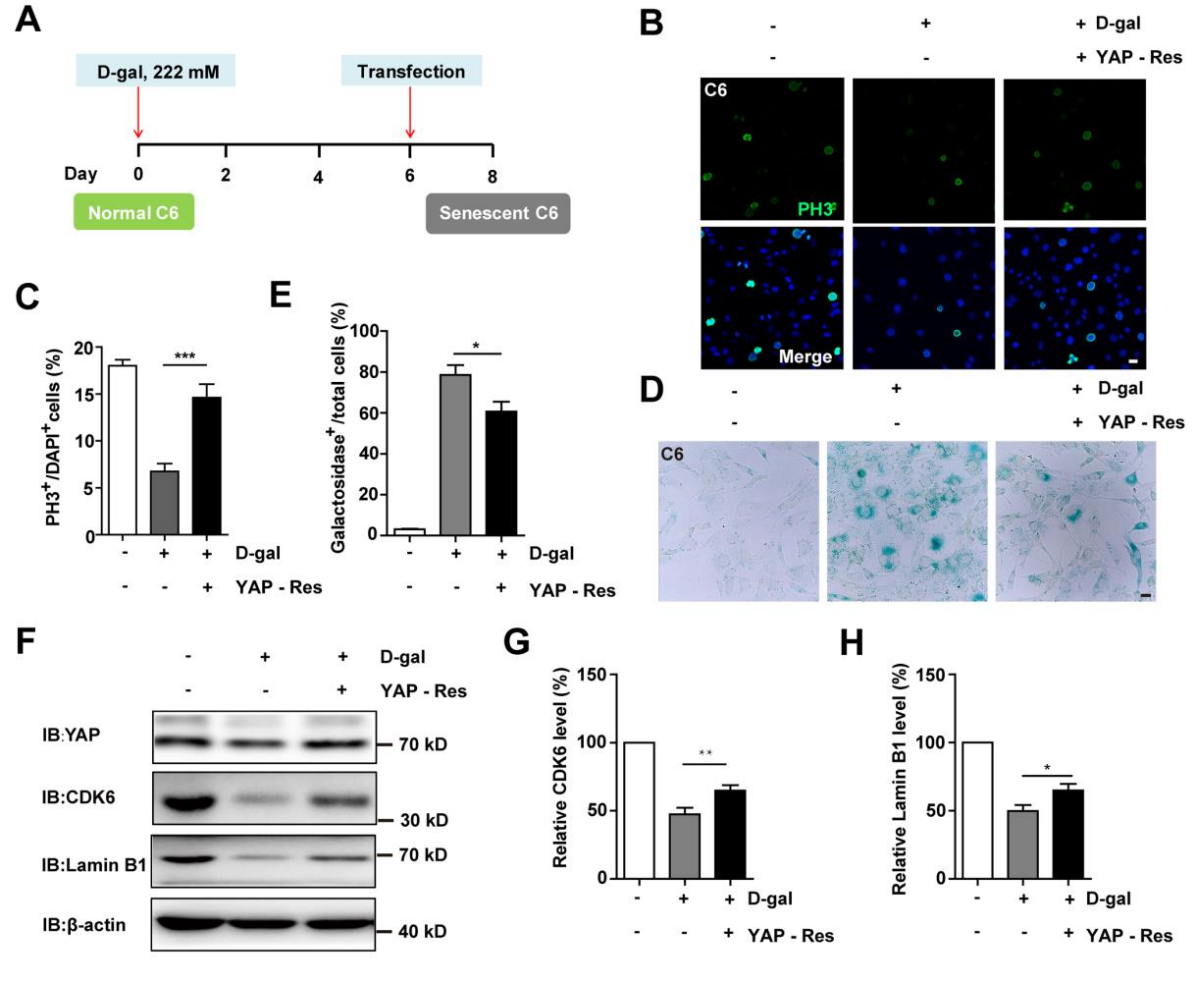
**Overexpression of YAP restored D-gal-induced GBM senescence.** (**A**) A schematic illustration showing YAP transfection in C6 senescent cells. (**B**) Immunostaining of PH3 in control C6 cells without transfection, or senescent C6 cells (treated with 222 mM D-gal for 8 d) transfected with EGFP or YAP-EGFP plasmid (YAP-Res) for 2 d. (**C**) Quantitative analysis of the percentage of PH3^+^ cells over total C6 cells as shown in (**B**) (n=15). (**D**) Representative images showing β-galactosidase staining in control C6 cells without transfection, or senescent C6 cells (treated with 222 mM D-gal for 8 d) transfected with EGFP or YAP-EGFP plasmid (YAP-Res) for 2 d. (**E**) Quantification of the percentage of β-galactosidase^+^ cells over total cells as shown in (**D**) (n=15). (**F**) Western blot detected the expression of YAP, CDK6, and Lamin B1 in control C6 cells without transfection, or senescent C6 cells (treated with 222 mM D-gal for 8 d) transfected with EGFP or YAP-EGFP plasmid (YAP-Res) for 2 d. (**G**, **H**) Quantification of CDK6 and Lamin B1 level as shown in (**F**) (n=10). Scale bars, 20 μm. Data shown are mean ± s.e.m. ^*^P < 0.05, ^**^P < 0.01. ^***^P < 0.001.

Previous studies have shown that inhibition of the YAP-CDK6 signaling pathway promotes the senescence of human fibroblasts [24]. Based on this, we tested whether this pathway played a role in D-gal-induced senescence of GBM cells. As expected, the protein level of LATS1, YAP, CDK6, and Lamin B1 were decreased, while the expression of p-YAP/YAP, p-MST1/MST1, p-LATS1/LATS1, and p27^kip1^ (a cyclin-dependent kinases inhibitor, which negatively regulates the cell cycle) were significantly elevated in D-gal-induced senescent C6 cells (Figure 6A–6I). These results suggest that the YAP-CDK6 pathway may be involved in GBM cell senescence in a Hippo kinase dependent manner. To further test this hypothesis, YAP was overexpressed in senescent C6 cells, in order to rescue D-gal-induced senescence (Figure 7A). Overexpression of YAP indeed restored cell proliferation (Figure 7B, 7C), as well as restored D-gal-induced senescence of C6 cells (Figure 7D, 7E). Moreover, overexpression of YAP also significantly restored D-gal-induced decrease of CDK6 and Lamin B1 expression (Figure 7F–7H), suggesting that D-gal might induce senescence of GBM cells through inactivating the YAP-CDK6 pathway.

**Figure 8:**
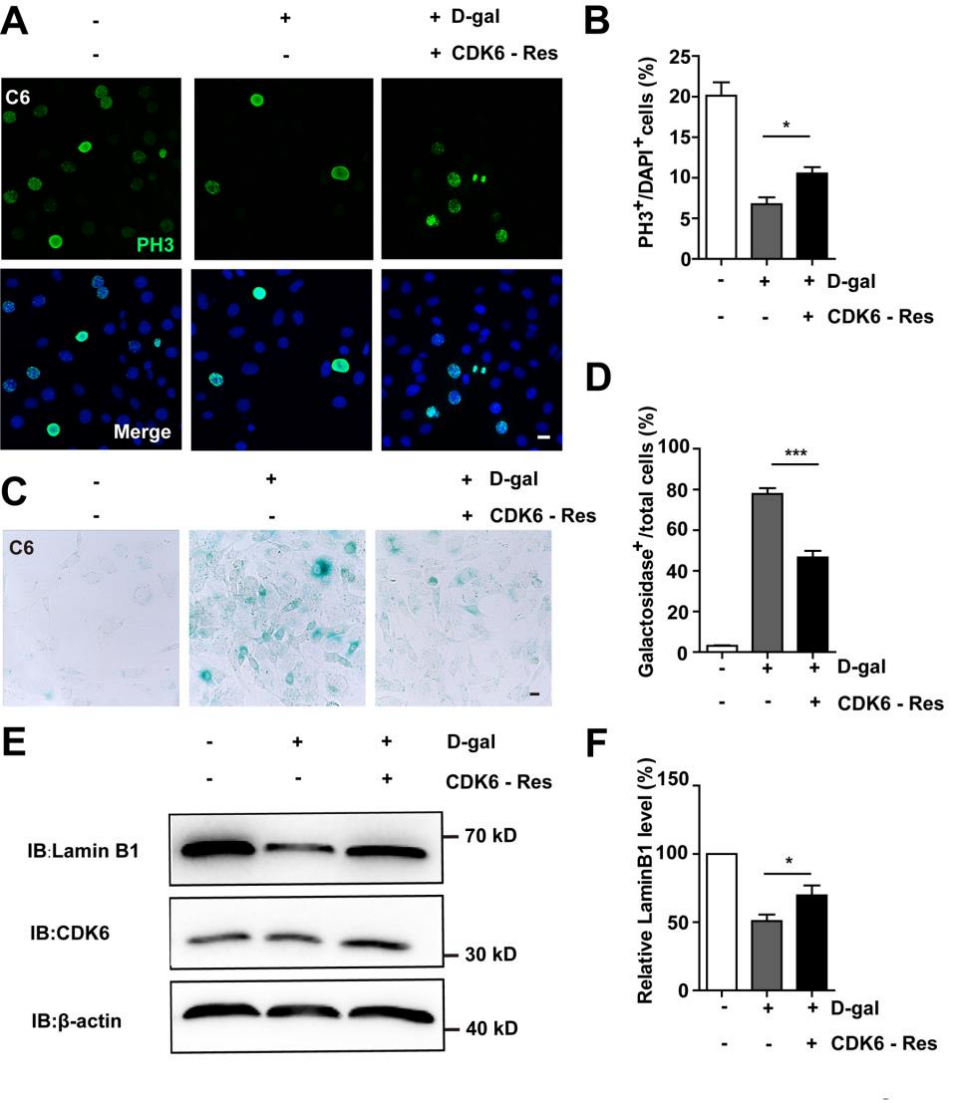
**Overexpression of CDK6 restored D-gal-induced GBM senescence.** (**A**) Immunostaining of PH3 in control C6 cells without transfection, or senescent C6 cells (treated with 222 mM D-gal for 8 d) transfected with pcDNA3.1 or pcDNA3.1-CDK6 plasmid (CDK6-Res) for 2 d. (**B**) Quantitative analysis of the percentage of PH3^+^ cells over total C6 cells as shown in (A) (n=15). (**C**) Representative images showing β-galactosidase staining in control C6 cells without transfection, or senescent C6 cells (treated with 222 mM D-gal for 8 d) transfected with pcDNA3.1 or pcDNA3.1-CDK6 plasmid (CDK6-Res) for 2 d. (**D**) Quantification of the percentage of β-galactosidase^+^ cells over total cells as shown in (**C**) (n=15). (**E**) Western blot detected the expression of CDK6, and Lamin B1 in control C6 cells without transfection, or senescent C6 cells (treated with 222 mM D-gal for 8 d) transfected with pcDNA3.1 or pcDNA3.1-CDK6 plasmid (CDK6-Res) for 2 d. (**F**) Quantification of Lamin B1 level as shown in (**E**) (n=10). Scale bars, 20 μm. Data shown are mean ± s.e.m. ^*^P < 0.05, ^***^P < 0.001.

Then CDK6 rescue experiments were performed. Overexpression of CDK6 indeed restored cell proliferation (Figure 8A, 8B), as well as restored D-gal-induced senescence of C6 cells (Figure 8C–8D). Moreover, overexpression of CDK6 also significantly restored D-gal-induced decrease in Lamin B1 expression (Figure 8E, 8F). Taken together, these results suggest that D-gal induces senescence of GBM cells by inactivating the YAP-CDK6 pathway.

### Metformin reverses D-gal-induced senescence of GBM cells by activating the YAP-CDK6 pathway

**Figure 9:**
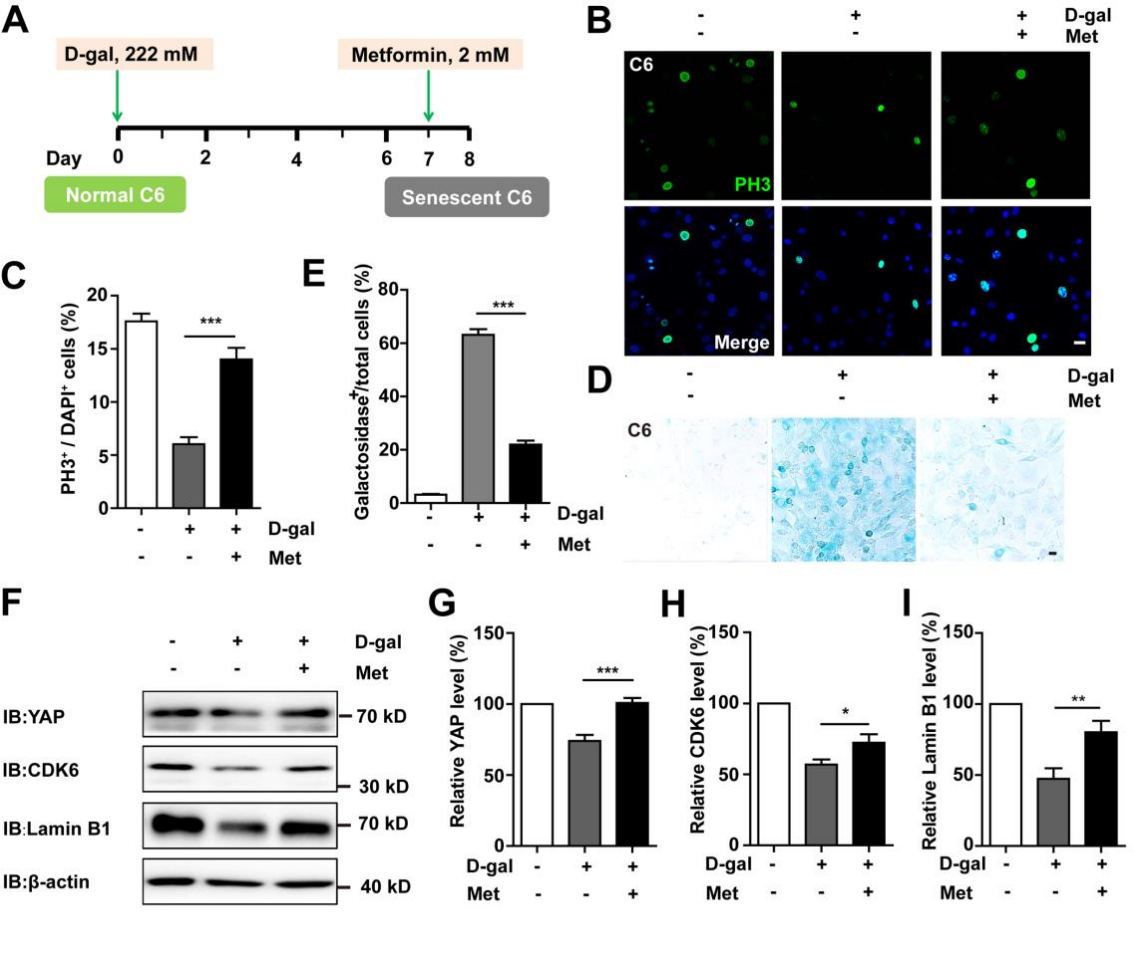
**Metformin reversed D-gal-induced senescence of GBM cells by activating YAP-CDK6 pathway.** (**A**) A schematic illustration showing metformin treatment in the presence of D-gal. (**B**) Immunostaining of PH3 in control C6 cells, or senescent C6 cells (treated with 222 mM D-gal for 8 d), or senescent C6 cells (treated with 222 mM D-gal for 8 d) with metformin treatment (2 mM for 1 d). (**C**) Quantitative analysis of the percentage of PH3^+^ cells over total C6 cells as shown in (**B**) (n=15). (**D**) Representative images showing β-galactosidase staining in control C6 cells, senescent C6 cells (treated with 222 mM D-gal for 8 d), or senescent C6 cells (treated with 222 mM D-gal for 8 d) with metformin treatment (2 mM for 1 d). (**E**) Quantification of the percentage of β-galactosidase^+^ cells over total cells as shown in (**D**) (n=15). (**F**) Western blot detected the expression of YAP, CDK6, and Lamin B1 in control C6 cells, or senescent C6 cells (treated with 222 mM D-gal for 8 d), or senescent C6 cells (treated with 222 mM D-gal for 8 d) with metformin treatment (2 mM for 1 d). (**G**–**I**) Quantification of YAP (n=6), CDK6 (n=15), and Lamin B1 (n=8) level as shown in (**F**). Met: metformin. Scale bars, 20 μm. Data shown are mean ± s.e.m. ^*^P < 0.05, ^**^P < 0.01, ^***^P < 0.001.

To determine whether D-gal-induced GBM cell senescence can be reversed, metformin, a promising anti-ageing agent [27], was applied in the senescent C6 cells (Figure 9A). As expected, metformin significantly restored cell proliferation (Figure 9B, 9C) as well as ameliorated D-gal-induced senescence of C6 cells (Figure 9D, 9E). Interestingly, metformin treatment also significantly restored D-gal-induced reduction in the expression of YAP, CDK6, and Lamin B1 (Figure 9F–9I). Taken together, these results suggest that metformin could reverse D-gal-induced GBM cell senescence by activating the YAP-CDK6 signaling pathway.

## DISCUSSION

**Figure 10:**
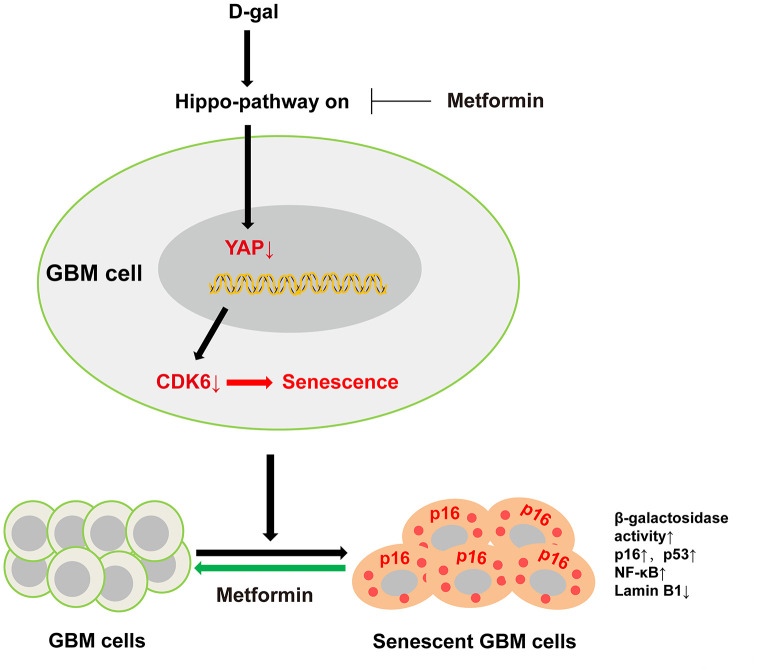
**A working model of D-gal-induced senescence of GBM cells.** D-gal treatment inactivates YAP-CDK6 pathway in GBM cells, inducing the characteristics of senescent cells, such as reduced cell proliferation, hypertrophic morphology, increased activity of senescence-associated β-galactosidase, and upregulation of several senescence-associated genes such as p16, p53, and NF-κB, and downregulation of Lamin B1. Metformin, an anti-ageing agent, reverses D-gal-induced senescence of GBM cells.

In the present study, we successfully established a new method for inducing GBM cell senescence using D-gal, and revealed that D-gal induced GBM cell senescence through the YAP-CDK6 signaling pathway. Moreover, our results show that metformin, an anti-ageing agent, can effectively reverse D-gal-induced senescence in GBM cells (Figure 10). This method can be utilized to explore the underlying mechanisms of GBM cell senescence, and to screen promising anti-aging drugs for GBM treatment.

D-gal is a simple, convenient, low cost agent that exhibits stable results, and has been used to induce senescence of different types of cells and ageing in animals [17–20]. The common phenotypes of cellular senescence include an enlarged, flattened, and vacuolized cell morphology, elevated senescence-associated β-galactosidase activity, attenuated cell proliferation, and accumulation of p16 [19, 20]. In this study, our findings indicated that D-gal-induced GBM cell senescence exhibited almost all the aforementioned features, which was consistent with these previous studies, suggesting that our model was successful. Our results revealed that, D-gal induced senescence of two GBM cell lines (C6 and U87MG), but not the neuroblastoma SH-SY5Y cell line. C6 cells originate from glial cells of rat brain, U87MG cells are derived from the human brain, while SH-SY5Y cells originate from the human bone marrow. Different sensitivities to environmental changes and different properties of these cells [28, 29] might help to explain why C6 and U87MG cells show different sensitivities to D-gal treatment in our study, compared to SH-SY5Y cells.

Reports have indicated that YAP inhibits cell apoptosis through activation of survival signaling, while phosphorylation is the main pathway for inhibiting YAP activity [30–32]. However, in our study, D-gal treatment didn’t cause apparent cell apoptosis, instead, it inhibited cell apoptosis, although increased p-YAP expression was found, which was surprising. Senescent cells have been reported to exhibit resistance to apoptosis, which is also one of the features of senescence [33]. Moreover, previous studies have shown that with pro-senescent stresses, p53 accumulates but to a lesser extent than during apoptosis, and this leads to less significant levels of pro-apoptotic p53-upregulated modulators of apoptosis [34]. Therefore, although D-gal treatment increased the expression of p-YAP and p53, this might not be sufficient to activate the apoptotic pathway and trigger cell apoptosis. In future, further analysis of the proteins involved in the apoptotic pathway is required.

Hypertonic stress represents an immense threat to most human cells. It has been reported that 225mM D-gal reduces the viability of human lens epithelium cells (LECs) to almost 0% after only 48h [20]. However, the same concentration may result in different effects for different cells. Actually, D-gal was used to induce senescence of non-cancer cells such as astrocytes, lens epithelium cells, and the optimal concentration was 55 mM and 125 mM, respectively, indicating that for different cell types, the concentration of D-gal to be used to induce cellular senescence may be different [19, 20]. GBM cells are different cell types from astrocytes or lens epithelium cells. There is evidence showing that hypertonicity induces swelling of human fibroblasts, due to unbalanced growth, thereby leading to cellular senescence [35]. In our study, D-gal at different concentrations was used to induce senescence, but only treatment using 222 mM for 8 days could induce their senescence. Under this concentration, however, few cases of cell death in C6 and U87MG cells were observed, and cells became larger, suggesting that GBM cells might overcome the hypertonic stress (222 mM D-gal), probably through inducing GBM cells into cellular senescence state. Moreover, Na Li and the colleagues found that D-gal at the concentrations higher than 30 g/L (167 mM) inhibited the viability of malignant cell lines, such as N2a, PC-3 and HepG2 cells in a dose-dependent manner, but NIH3T3 cells and LO_2_ cells (both non-malignant cells) were not sensitive to D-gal treatment [36], indicating that at the same concentration of D-gal, some cell types might be more tolerant. Furthermore, the results also showed that up to 60 g/L (333 mM, with hypertonicity) of glucose (with the same molecular formula and molecular weight of D-gal) did not inhibit the viability of malignant cell lines significantly, indicating that some malignant cell lines can overcome higher hypertonic stress (333 mM glucose) [36]. Therefore, GBM cells might overcome hypertonic stress (222 mM D-gal) through unknown mechanisms, maybe by inducing senescence of GBM cells. The detailed mechanisms require further investigation in the future.

Various drugs, apart from, D-gal-induced GBM cell senescence model, have been found to induce *in vitro* senescence of GBM cells. These include berberine [37], flavokawain B (FKB), a natural kava chalcone [38], and matrine [39]. However, different cell senescence models show different disadvantages. For example, etoposide, a DNA-damaging drug, can be used to prepare a senescence model of tumor cells [26, 40], but the compound is highly toxic and triggers apparent cell apoptosis when used at high concentrations [41]. In addition, human lung fibroblast, IMR-90 cell, is almost completely senescent when it passes through 50 generations [24]. However, this method is expensive, consumes a lot of time, and is labor-consuming. Our findings suggested that D-gal could induce senescence of GBM cells in a method that is simple, fast, and with high success rate as well as good effects.

The mechanisms underlying the senescence of GBM cells may vary under different conditions [37, 39, 42]. For example, berberine induces senescence of GBM cells by downregulating the EGFR-MEK-ERK signaling pathway [37]. On the other hand, flavokawain B induces senescence of these cells via endoplasmic reticulum stress-induced autophagy [38], whereas matrine has been found to induce senescence of human GBM cells through suppression of the IGF1/PI3K/AKT/p27 signaling pathway [39].

Although the mechanism underlying D-gal-induced aging in animals is still not fully understood, the mainstream view is that D-gal produces aldose and hydrogen peroxide, under the action of galactose oxidase. This increases reactive oxygen species (ROS), lipid peroxidation, and produces superoxide anion free radicals, leading to ageing of the body [17]. In addition, some studies have reported that the concentration of galactose in the cells increases after continuous D-gal injection, and the galactose is transformed to galactitol by the galactose reductase, which can’t be further metabolized. Consequently, accumulation of galactitol in cells increases osmotic pressure, leading to swelling of the cells and dysfunction, eventually causing ageing [17, 43]. At the molecular level, D-gal induces premature senescence of lens epithelial cells by disturbing autophagy flux and mitochondrial functions [20], or induces astrocytes senescence through glutamine synthetase signaling [19]. Based on the results of the current study, several lines of evidence suggest that the YAP-CDK6 signaling pathway mediated D-gal-induced senescence of GBM cells. Firstly, D-gal treatment inactivated YAP and CDK6 in GBM cells. Secondly, overexpression of YAP or CDK6 restored D-gal-induced senescence of GBM cells. Finally, metformin, a potential anti-aging agent, activated the YAP-CDK6 pathway and suppressed D-gal-induced senescence of C6 cells. Our implication of the YAP-CDK6 pathway in senescence is in line with recent observations that have demonstrated that YAP controls senescence of IMR90 cells [24], human mesenchymal stem cells [44], hepatocytes [45], and YAP-1 deficiency promotes health ageing of *Caenorhabditis elegans* [46]. Moreover, some other YAP-targeted genes, such as CYR61 (Cysteine Rich Angiogenic Inducer 61) and CTGF (Connective Tissue Growth Factor), are also reported to be involved in cellular senescence [47, 48]. Therefore, we tested the mRNA level of CYR61 and CTGF, and found that YAP decreased the mRNA level of CYR61, while the mRNA level of CTGF was significantly increased (Supplementary Figure 4), indicating that other YAP-targeted genes might also be involved in D-gal-induced senescence of GBM cells. Whether CYR61 is involved in YAP-inhibited glioma senescence, further researches are needed.

Metformin, an oral hypoglycemic agent used since the 1960’s for treatment of type 2 diabetes and metabolic syndrome, ameliorates the physical condition and cognitive function in neurodegenerative disease-related models [49]. In addition, it improves health and prolongs lifespan of mice [27], *Caenorhabditis elegans* [50], as well as humans [51, 52]. In this regard, metformin is believed to be an effective anti-ageing agent. The mechanism underlying the anti-ageing effects of metformin involves, at least partially, activation of AMPK [27]. Moreover, studies have reported that metformin functions through AMPK-independent mechanisms [53]. Recently, some researches have demonstrated the ability of metformin to exert anti-cancer effects by inhibiting the function of YAP [54–56]. In our study, we found that metformin inhibited senescence of GBM cells by activating the YAP-CDK6 signaling pathway and inhibiting cell proliferation, a new mechanism to be implicated in GBM cell senescence. It is possible that this compound could play different roles under cancer or cell senescence conditions although this remains to be studied further.

Our D-gal-induced GBM cell senescence model also has limitations. First, D-gal-induced cell senescence is not applicable to all types of cells. For example, the findings of this study indicated that D-gal could induce senescence in only C6 and U87MG GBM cells, but not SH-SY5Y cells. Secondly, this method can also induce cell senescence in normal cells, such as astrocytes [19]. It is possible that some side-effects may appear when D-gal is applied *in vivo*. Therefore, a targeted strategy, such as local application of D-gal may be required before its application. In the future, an initial screening of drugs that can selectively induce senescence of GBM cells followed by those that can kill these cells might be an effective and promising strategy. In fact, researchers have developed such a strategy that is effective in managing liver cancer in animal models. Specifically, CDC7 inhibitors have first been used to induce senescence of hepatocellular carcinoma cells, followed by sertraline application to induce apoptosis of the senescent cells. Such a combined therapy was found to significantly inhibit progression of liver cancer [57]. However, GBM is much more difficult to be cured, compared to other types of tumors, therefore, whether this combined therapy is applicable to GBM requires further investigation.

In summary, our findings not only established a new model for GBM cell senescence, but also identified the YAP-CDK6 pathway as the mechanism for D-gal-induced senescence of GBM cells, which can provide a new model basis and novel insights for the treatment of GBM.

## MATERIALS AND METHODS

### cDNA constructs

We used pEGFP, pEGFP-hYAP1, pcDNA3.1, and pcDNA3.1-CDK6 cDNA vectors for expression and rescue experiments. pEGFP-hYAP1 (http://www.addgene.org/17843) and pcDNA3.1-CDK6 (http://www.addgene.org/75170/) were purchased from Addgene. All these expression constructs were verified by sequencing.

### Cell culture

Glioma cell lines, C6, SH-SY5Y, and U87MG were kindly provided by Prof. Maojin Yao (Sun Yat-Sen University), and were maintained in Dulbecco’s Modified Eagle’s Medium/Nutrient Mixture F-12 (Gibco), supplemented with 10% FBS (Gibco) and 1% penicillin/streptomycin (Gibco), under a humidified atmosphere of 5% CO_2_ at 37°C.

### Cell viability assay

Cell viability was measured using the CCK-8 cell counting kit (A311-01/02, Vazyme Biotech). Briefly, for cells involved in D-gal experiments, the cells were first cultured in D-gal-containing medium for 6-7 days in 35 mm dishes, then seeded into 96-well plates (in D-gal-containing medium) at a density of approximately 2, 000 cells/well and cultured for 24-48 h. For cells involved in etoposide experiments, cells were first treated with 12 μM etoposide for 1 day, and recovered for 2-3 day (without etoposide treatment) in 35 mm dishes, then seeded into 96-well plates (without etoposide treatment) at a density of approximately 2, 000 cells/well and cultured for 24-48 h. Subsequently, 10 μL CCK-8 solution was added to each well and incubated at 37°C for 2 h. An optical density at 450 nm, which indicates a positive correlation with cell viability, was measured using a microplate reader (Varioskan Flash, Thermo scientific, USA). The optical density of the each group was normalized to the average optical density of the control group.

### Establishment of GBM cell senescence models

The senescence of C6 cells was induced by D-gal (D8310, Solarbio) treatment at 222 mM for 8 days, or by etoposide (E1383, Sigma) at 6 μM for 1 day, then recovered after a further 4 days. The senescence of U87MG cells was induced by D-gal treatment at 222 mM for 7 days. To rescue D-gal-induced glioma cell senescence, metformin (HY-17471A, MedChemExpress) was dissolved in water (1 M in stock solution) and applied at the concentration of 2 mM for 1 day.

### Senescence-associated β-galactosidase staining

β-galactosidase staining kit (G1580, Solarbio) was used to evaluate the senescence of C6 or U87MG or SH-SY5Y cells. Summarily, the following steps were performed: Cells were first washed twice with HBSS and fixed with β-galactosidase fixative solution at room temperature for 15 min. The cells were then washed 3 times with HBSS, for 3 min each time, followed by addition of 1 mL dyeing liquid (10 μL β-galactosidase staining fluid A, 10 μL fluid B, 930 μL fluid C, and 50 μL X-Gal solution), and incubated at 37°C overnight. The cells were finally observed under an inverted microscope. Fine blue uniform particles in the cytoplasm considered β-galactosidase positive signals.

### Western blot analysis

Western blot analysis was carried out as previously described [58]. Briefly, C6 or U87MG cells were lysed by ice-cold RIPA Buffer (P0013B, Beyotime) and incubated at 4 °C for 30 min. Proteins were extracted using 5× loading buffer, following centrifugation at 12, 000 ×g for 30 min, and boiled at 100°C for 8-10 min. The proteins were then separated using 10-12% sodium dodecyl sulfate-polyacrylamide gel electrophoresis (SDS-PAGE) and transferred into nitrocellulose membranes (Life sciences, USA). After blocking in TBST containing 5% skimmed milk for 1 h, the immunoblots were incubated at 4°C overnight with different primary antibodies. Primary antibodies used included rabbit anti-caspase-3 (#13008, CST, 1:1, 000), rabbit anti-cleaved caspase-3 (#9579, CST, 1:1, 000), rabbit anti-Lamin B1 (ab16048, Abcam, 1 : 1, 000), rabbit anti-p53(bs-2090R, Bioss, 1:1,000), rabbit anti-NF-κB (ab16502, Abcam, 1 : 1, 000), mouse anti-YAP (WH0010413M1, Sigma-Aldrich, 1:1, 000), rabbit anti-p-YAP (#13008, CST, 1:1, 000), rabbit anti-LATS1 (#3477, CST, 1:1000), rabbit anti-p-LATS1 (ser909) (#9157, CST, 1:1000), rabbit anti-MST1 (#3682, CST, 1:1000), rabbit anti-p-MST1/2 (Thr183/Thr180) (#49332, CST,1:1, 000), mouse anti-CDK6 (#3136T, CST, 1:1,000), and mouse anti-p27^kip1^ (#3698, CST, 1:1, 000). Rabbit anti-GAPDH (#2118, CST, 1:5, 000) or mouse anti-*β*-actin (A5316, Sigma-Aldrich, WB 1:10, 000) as a loading control was detected alongside the experimental samples. The membranes were subsequently washed three times in TBST, and incubated with the horseradish peroxidase (HRP) conjugated secondary antibodies for 1 h. After a further 3-time wash in TBST, the protein signals were detected using the ECL detection kit (Bio-Rad, USA) and analyzed using Quantity One software (Bio-Rad).

### RNA extraction and quantitative real-time PCR (qPCR)

To determine mRNA expression levels, total RNA was extracted from cells using the TRIzol^TM^ reagent (#15596026, Ambion) according to the manufacture’s protocol. A total of 2 μg RNA was reverse transcribed into cDNA using the SuperScript™ One-Step Reverse Transcription Kit (#10928-034, Invitrogen). Expression levels of mRNA were quantified using the iTaq™ Universal SYBR® Green Supermix (#172-5122, Bio-Rad) on the real-time PCR detection System (Applied Biosystems, USA). *β*-actin or GAPDH was chosen as the endogenous amplification controls. The relative expression levels were presented as ΔCt = Ct gene-Ct reference, and fold changes of gene expression were calculated using the 2^-ΔΔCt^ method. The following primers were synthesized by Sangon Biotech and used for amplification: *p16*, 5’-TGCAGATAGACTAGCCAGGGGA-3’ and 5’-CTTCCAGCAGTGCCCGCA-3’ [59]; *NF-κB*, 5’-CATCCACCTTCATGCTCAGC-3’ and 5’- CCACCACATCTTCCTGCTTG-3’[60]; *CYR61*, 5’-CTCAACGAGGACTGCAGCAA-3’ and 5’-CAGGGTCTGCCTTCTGACTGA-3’[61]; *CTGF*, 5’-CCGCCAACCGCAAGATT-3’ and 5’-CACGGACCCACCGAAGAC-3’[62]; *β*-actin, 5’-AAGTCCCTCACCCTCCCAAAAG-3’ and 5’- AAGCAATGCTGTCACCTTCCC-3’ [63]; *GAPDH*, 5’-AACTTTGGCATCGTGGAAGGG-3’ and 5’- AGGGATGATGTTCTGGGCTGC-3’[64].

### Immunocytochemistry

Protocols used for immunofluorescence staining and quantitative analysis were as previously described [11]. Briefly, cells were rinsed once with PBS, and fixed in 4% paraformaldehyde for 20 min. Then, they were blocked and permeabilized with 0.1% Triton X-100 in PBS containing 5% bovine serum albumin (BSA) at room temperature for 1 h. Thereafter, the cells were incubated with primary antibodies overnight, at 4 °C, washed 3 times in PBS and then incubated with secondary antibodies at room temperature for 1 h. The primary antibodies included rabbit anti-cleaved caspase-3 (#9579, CST, 1:200), rabbit anti-Lamin B1 (ab16048, Abcam, 1 : 200), mouse anti-PH3 (ab14955, Abcam, 1:2, 500), rabbit anti-PH3 (#06-570, Millipore, 1:200), and rabbit anti-Ki67 (AB9260, Millipore, 1:200). After washing in PBS for a further 3 times, cells were mounted. Images were acquired by using a fluorescence microscope (NIKON, Tokyo, Japan), and was analyzed by using Image J software.

### Statistical analysis

Statistical analyses were performed using GraphPad Prism 7.0 software with all data, derived from at least three independent experiments, expressed as mean ± standard error of the mean (s.e.m). For comparison between two groups involving one variate, we used an unpaired *t*-test. On the other hand, we employed a one-way analysis of variance (ANOVA) for comparisons between more than two groups involving one variate. For data involving two variates, a two-way ANOVA test was performed. A *P* value of < 0.05 was considered to be statistically significant.

## Supplementary Material

Supplementary Figures
