# Correction for: D-galactose induces senescence of glioblastoma cells through YAP-CDK6 pathway

**DOI:** 10.18632/aging.206264

**Published:** 2025-05-31

**Authors:** Xingxing Xu, Xiya Shen, Wenjin Feng, Danlu Yang, Lingting Jin, Jiaojiao Wang, Mianxian Wang, Zhang Ting, Feng Xue, Jingjing Zhang, Chaobo Meng, Roumeng Chen, Xinru Zheng, Leilei Du, Lina Xuan, Ying Wang, Tian Xie, Zhihui Huang

**Affiliations:** 1 School of Basic Medical Sciences, Wenzhou Medical University, Wenzhou 325035, Zhejiang, China; 2 Key Laboratory of Elemene Anti-Cancer Medicine of Zhejiang Province and Holistic Integrative Pharmacy Institutes, and Department of Neurosurgery of Affiliated Hospital, Hangzhou Normal University, Hangzhou 311121, China; 3 Department of Orthopedics (Spine Surgery), The First Affiliated Hospital of Wenzhou Medical University, Wenzhou 325035, Zhejiang, China; 4 Zhejiang Sinogen Medical Equipment Co., Ltd., Wenzhou 325000, Zhejiang, China; 5 Department of Transfusion Medicine, Zhejiang Provincial People’s Hospital of Hangzhou Medical College, Hangzhou 310053, China; 6 Department of Neurobiology, Key Laboratory of Medical Neurobiology, Ministry of Health of China, School of Medicine, Zhejiang University, Hangzhou 310058, China

**Keywords:** D-galatose, cellular senescence, glioblastoma, YAP, CDK6

**This article has been corrected:** The authors have identified the errors in the affiliations listed for Tian Xie. In the affiliation 2 the "Department of Neurosurgery of Affiliated Hospital" was removed:

^2^Key Laboratory of Elemene Anti-Cancer Medicine of Zhejiang Province and Holistic Integrative Pharmacy Institutes, Hangzhou Normal University, Hangzhou 311121, China

The affiliation 3 was also present and has now been removed.

Xingxing Xu^1,2,3,*^, Xiya Shen^1,2,3,*^, Wenjin Feng^4,*^, Danlu Yang^1,2,3^, Lingting Jin^1,2,3^, Jiaojiao Wang^1,2,3^, Mianxian Wang^1,2,3^, Zhang Ting^6^, Feng Xue^1^, Jingjing Zhang^1,2,3^, Chaobo Meng^1^, Roumeng Chen^1^, Xinru Zheng^1^, Leilei Du^1^, Lina Xuan^1^, Ying Wang^5^, Tian Xie^2^, Zhihui Huang^1,2,3^

^1^School of Basic Medical Sciences, Wenzhou Medical University, Wenzhou 325035, Zhejiang, China

^2^Key Laboratory of Elemene Anti-Cancer Medicine of Zhejiang Province and Holistic Integrative Pharmacy Institutes, Hangzhou Normal University, Hangzhou 311121, China

^3^Department of Orthopedics (Spine Surgery), The First Affiliated Hospital of Wenzhou Medical University, Wenzhou 325035, Zhejiang, China

^4^Zhejiang Sinogen Medical Equipment Co., Ltd., Wenzhou 325000, Zhejiang, China

^5^Department of Transfusion Medicine, Zhejiang Provincial People’s Hospital of Hangzhou Medical College, Hangzhou 310053, China

^6^Department of Neurobiology, Key Laboratory of Medical Neurobiology, Ministry of Health of China, School of Medicine, Zhejiang University, Hangzhou 310058, China

^*^Equal contribution

Additionally, the authors have identified an inadvertent error in the organization of Figure 7F. Due to the high similarity between the Western Blot raw data bands for CDK6 and Lamin B1, the Lamin B1 band in Figure 7F was mistakenly represented using the same raw band as CDK6. The authors have replaced the incorrect image with the correct original image for Lamin B1. They have also stated that this correction does not affect the experimental outcome or the conclusions of the study and sincerely apologize for this error and any inconvenience it may have caused.

The corrected version of **Figure 7** is provided below.

**Figure 7:**
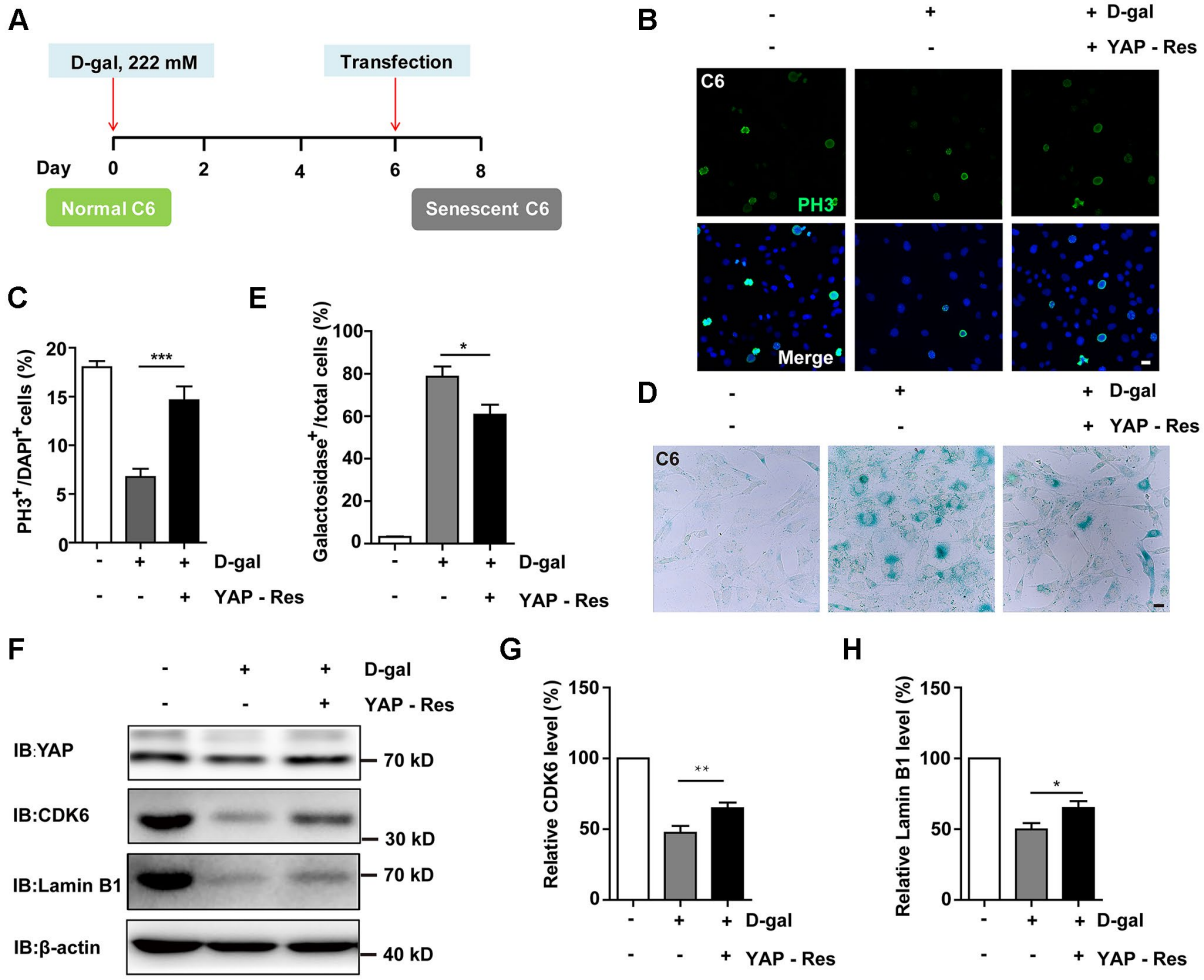
**Overexpression of YAP restored D-gal-induced GBM senescence.** (**A**) A schematic illustration showing YAP transfection in C6 senescent cells. (**B**) Immunostaining of PH3 in control C6 cells without transfection, or senescent C6 cells (treated with 222 mM D-gal for 8 d) transfected with EGFP or YAP-EGFP plasmid (YAP-Res) for 2 d. (**C**) Quantitative analysis of the percentage of PH3^+ ^cells over total C6 cells as shown in (**B**) (*n* = 15). (**D**) Representative images showing β-galactosidase staining in control C6 cells without transfection, or senescent C6 cells (treated with 222 mM D-gal for 8 d) transfected with EGFP or YAP-EGFP plasmid (YAP-Res) for 2 d. (**E**) Quantification of the percentage of β-galactosidase^+^ cells over total cells as shown in (**D**) (*n* = 15). (**F**) Western blot detected the expression of YAP, CDK6, and Lamin B1 in control C6 cells without transfection, or senescent C6 cells (treated with 222 mM D-gal for 8 d) transfected with EGFP or YAP-EGFP plasmid (YAP-Res) for 2 d. (**G**, **H**) Quantification of CDK6 and Lamin B1 level as shown in (**F**) (*n* = 10). Scale bars, 20 μm. Data shown are mean ± s.e.m. ^*^P < 0.05, ^**^P < 0.01. ^***^P < 0.001.

