# Supplementary material for: D-galactose induces senescence of glioblastoma cells through YAP-CDK6 pathway
Source: Aging (Albany NY). 2020 Sep 29;12(18):18501–21. doi: 10.18632/aging.103819 (PMC7585072; doi:10.18632/aging.103819)
Supplement: Supplementary Figures [file aging-12-103819-s001..pdf]

SUPPLEMENTARY FIGURES

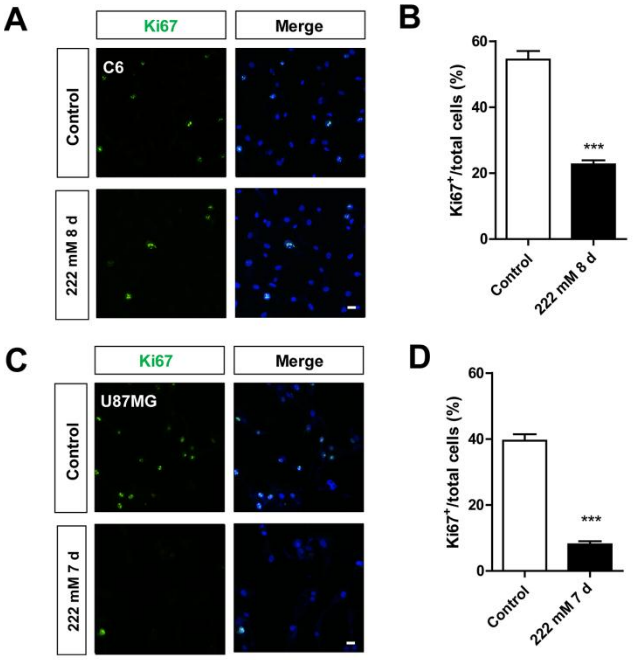

Supplementary Figure 1. D-gal inhibited the proliferation of C6 and U87MG cells. (**A**) Immunostaining of Ki67 (green) in control and senescent C6 cells (treated with 222 mM D-gal for 8 d). (**B**) Quantitative analysis of the percentage of $Ki67^+$ cells over total cells as shown in (**A**) (n=15). (**C**) Immunostaining of Ki67 (green) in control and senescent U87MG cells (treated with 222 mM D-gal for 7 d). (**D**) Quantitative analysis of the percentage of $Ki67^+$ cells over total cells as shown in (**C**) (n=15). Scale bars, 20 µm. Data shown are mean ± s.e.m. ****P < 0.001*.

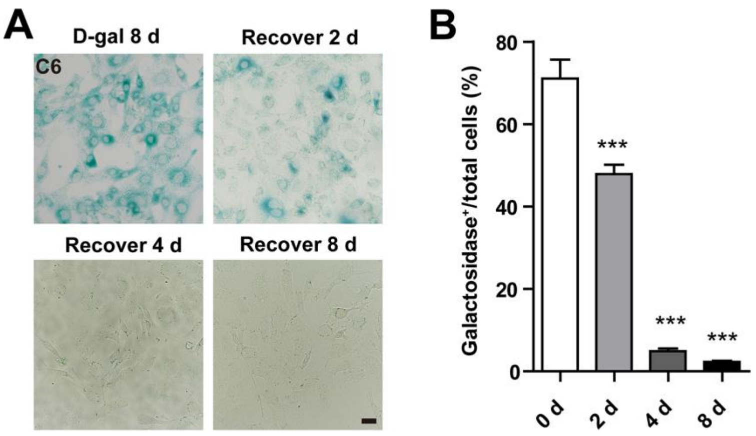

Supplementary Figure 2. D-gal-induced C6 cell senescence can be reversed. (**A**) Representative images showing β-galactosidase staining in senescent C6 cells (treated with D-gal at 222 mM for 8 d) and senescent C6 cells after removal of D-gal for 2, 4, 8 d. (**B**) Quantification of the percentage of β-galactosidase$^+$ C6 cells over total cells as shown in (**A**) (n=15). Scale bar, 20 μm. Data shown are mean ± s.e.m. ****P < 0.001*.

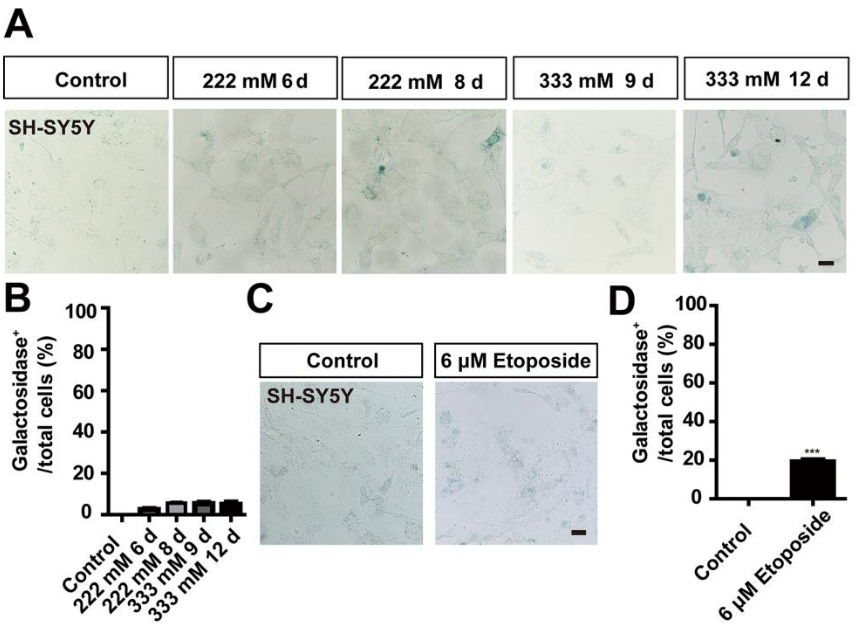

Supplementary Figure 3. D-gal failed to induce the senescence of SH-SY5Y cells. (**A**) Representative images showing β-galactosidase staining in control SH-SY5Y cells and SH-SY5Y cells treated with D-gal at 222 mM for 6 and 8 d, or 333 mM for 9 and 12 d. (**B**) Quantification of the percentage of β-galactosidase$^+$ SH-SY5Y cells over total cells as shown in (**A**) (n=15). (**C**) Representative images of β-galactosidase staining in control SH-SY5Y cells and SH-SY5Y cells treated with etoposide at 6 μM for 1 d, and recovered for 4 d. (**D**) Quantification of the percentage of β-galactosidase$^+$ SH-SY5Y cells over total cells as shown in (**C**) (n=15). Scale bars, 20 μm. Data shown are mean ± s.e.m. ****P < 0.001*.

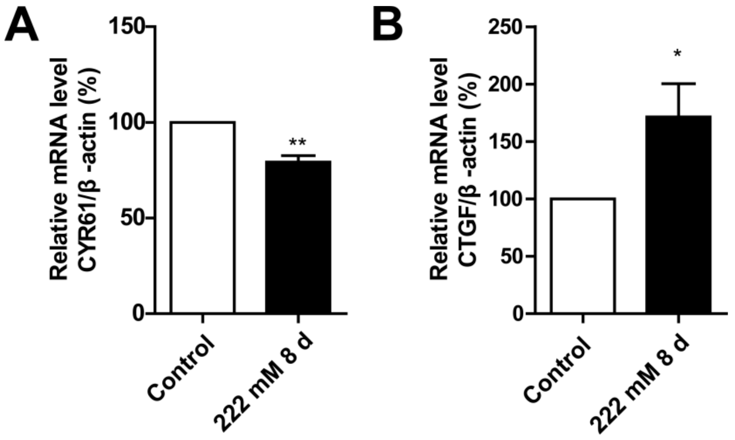

Supplementary Figure 4. D-gal treatment altered the expression of other YAP-targeted genes. (**A, B**) qPCR results showing the mRNA level of CYR61 (**A**, n=4) and CTGF (**B**, n=7) in control and senescent C6 cells (treated with 222 mM D-gal for 8 d). Data shown are mean ± s.e.m. $^{*}P < 0.05$, $^{**}P < 0.01$.
